# PROTOCOL: Cognitive and behavioral radicalization: A systematic review of the putative risk and protective factors

**DOI:** 10.1002/cl2.1102

**Published:** 2020-09-09

**Authors:** Michael Wolfowicz, Yael Litmanovitz, David Weisburd, Badi Hasisi

**Affiliations:** ^1^ Institute of Criminology, Faculty of Law The Hebrew University of Jerusalem, Mount Scopus Jerusalem Israel

## Abstract

**Objectives:**

This systematic review seeks to collate and synthesize putative risk and protective factors for the different outcomes of radicalization.

**Methodology:**

Drawing on an established theoretical framework, we will categorize putative risk and protective factors as they relate to the domains of radical attitudes, intentions, and behaviors. We will utilize meta‐analytic techniques to develop a rank‐order of factors according to effect size. Meta‐regression and sub‐group analyses will be used to assess sources of heterogeneity.

**Implications:**

The results of the review are intended to inform evidence‐based policy in the areas of both assessment and intervention.

## 1 BACKGROUND

### 1.1 The problem

Terrorism continues to be one of the most serious challenges in the world today, especially for democratic countries that have experienced an uptick in terrorism in recent times. This uptick has coincided with a growth in specific threats, such as homegrown terrorism, lone‐wolf terrorism, and the issue of departing and returning foreign fighters (Global Terrorism Index, 2016). Already beginning in about 15 years ago, a shift began to occur in which scholars and practitioners recognized that there was no profile of a terrorist, and that predicting terrorism was exceptionally difficult. It became clear that military might, intelligence, and policing alone are not capable of countering the multitude of threats posed by terrorism. Rather, an effective approach to combatting terrorism is one that balances active security with efforts to stymie those factors that lead to the radicalization that underpins terrorism (European Commission, 2014; OSCE, 2014; White House, 2011). Since there are far more radicalized individuals than there are terrorists, but almost all terrorists are radicalized, the new logic focused on reducing the prevalence of radicalization in the population as a means of reducing the risk of terrorism (European Commission, 2005a, 2014; OSCE, 2014; White House, 2011).

It is this logic that has given rise to an increased focus on efforts to improve risk assessment, and a host of counter‐radicalization and deradicalization strategies that seek to target the factors that increase risk, and promote factors that decrease it. Under the umbrella of counter violent extremism (CVE) policies, such strategies and initiatives seek to both predict future radical behavior, as well as change it. CVE strategies include a range of socio‐economic interventions, community and education initiatives, and online interventions. All of these approaches share the common underlying assumption and approach that by tackling the radicalization that underpins the likelihood of recruitment to terrorism, and by reducing the prevalence of radicalized individuals in the population, there will ultimately be a reduction in the likelihood of terrorism (EU, 2014; OSCE, 2014).

Currently, many of these initiatives are not evidence based. Despite the growth of radicalization and terrorism research in recent years, the empirical study still only accounts for a small percentage of the knowledge (Christmann, 2012; Lum, Kennedy, & Sherley, 2006; Schuurman, 2018; Silke, 2008). There is therefore little concrete information upon which policies and interventions can be developed, and they are therefore unlikely to have the desired impact (Davis, 2014). While such policies and strategies aim to tackle risk factors, there is often mixed and contradictory evidence about what the risk factors are, and their relative importance (Hafez & Mullins, 2015). The lack of systematic investigation has left it to policy makers to develop policies and strategies that are not evidence based (Neumann & Kleinmann, 2013; Victoroff, 2005). Only “Greater analytical depth may eventually reconcile contradictory claims" (Wikström & Bouhana, 2017, p. 183).

It is for these reasons that a systematic review and meta‐analysis are needed. While systematic reviews are intended to focus on specific and narrow research questions, previous systematic reviews on radicalization have been overly broad, in part because of a lack of a cohesive for conceptualizing radicalization. In this review, we take a more focused approach, enabling a meaningful synthesis and organization of quantifiable data, whilst maintaining a broad enough scope as to capture the complexity of the factors related to radicalization outcomes. To achieve this, we follow an established theoretical model for predetermining our outcomes of interest, namely different measures of cognitive and behavioral radicalization; the two‐pyramid model (TPM; McCauley & Moskalenko, 2017). Moreover, unlike previous reviews, the current study is limited to examining rigorous quantitative studies only, which provide sufficient data to calculate effect sizes for input into a meta‐analysis.

### 1.2 Defining radicalization and recruitment

The suffix of the word radicalization indicates that it is a process, something which all scholars agree can take place over a period of time from as short as a few weeks to as long as many years (Borum, 2011a, 2011b; Klausen, Libretti, Hung, & Jayasumana, 2018; Silke, 2008). But definitions of radicalization that focus too much on the process element can cause confusion, since they highlight violent extremism, or terrorism as being the ultimate outcome of this process. In fact, very few radicalized individuals will even go on to carry out any acts of violence. That is, only a small percentage of individuals who hold radical attitudes, arguably less than 1%, will ever engage in any form of radical behaviors (Borum, 2011a, 2011b; Horgan, 2008; McCauley & Moskalenko, 2017).

According to the EU, radicalization is defined as "the phenomenon of people embracing opinions, views and ideas which *could* [sic] lead to acts of terrorism” (European Commission, 2005a). As this definition indicates, there is a clear difference between the ideas, attitudes, and opinions that connote radicalization on one hand, and acts of terrorism on the other hand. Indeed, the EU has a separate definition for recruitment to terrorism, with recruitment being when someone has been solicited “to commit or participate in the commission of a terrorist offense, or to join an association or group, for the purpose of contributing to the commission of one or more terrorist offenses by the association or the group” (European Commission, 2005b), or in short, “Recruitment to carry out terrorist offenses” (European Commission, 2014). As such, anyone who has participated in a terrorist offense is said to have been recruited.

The EU's definitions for radicalization and recruitment underscore the ever‐important distinction that needs to be made between the cognitive and behavioral dimensions and outcomes of radicalization (Bartlett, Birdwell, & King, 2010; Borum, 2011a, 2011b; Hafez & Mullins, 2015; Khalil, 2014; P. R. Neumann, 2013; Vidino & Brandon, 2012). However, they do not indicate what types of ideas, attitudes, or opinions constitute radicalization. A variety of proxies have been used in the literature for radicalization, or extremism, including support for extreme right‐wing parties (Perry, Wikström, & Roman, 2018; Rydgren & Ruth, 2013); measuring personality traits, such as the authoritarian personality scales (RWA), or fundamentalism (Beller & Kröger, 2017; McCann, 2009; McCleary, Quillivan, Foster, & Williams, 2011); and measures of orthodoxy and religious fundamentalism (Slootman & Tillie, 2006). On their own, these are all considered poor proxies as they have a low degree of specificity with respect to the associated behavioral outcome of interest, namely terrorism (Koopmans, 2015; Mudde, 2004).

Indeed, in any research that seeks to explore the attitudinal or cognitive antecedents of a given behavior, it is important that they have a high level of specificity with reference to the behavioral outcome of interest (Ajzen & Fishbein, 1980;  Fishbein & Ajzen, 1975). This approach underpins McCauley and Moskalenko's "radical opinions" outcomes, in which radical opinions, or what we refer to as attitudes, constitute the support for, justification of, or a belief that there is a personal obligation toward the carrying out of radical violence and/or terrorism. Using constructs that are in line with such measures to assess cognitive radicalization has become the standard in research (e.g., K. Bhui, Warfa, & Jones, 2014; Doosje et al., 2016; Kruglanski, Jasko, Chernikova, & Milyavsky, 2018; McCauley & Moskalenko, 2008; Schmid, 2013; Webber et al., 2018) and policy (e.g. Agerschou, 2014).

#### 1.2.1 The two‐pyramid model

This systematic review follows this highly specific approach by utilizing the outcome‐based typology (attitudes‐intentions‐behavior) embodied in the increasingly popular TPM model of radicalization developed by McCauley and Moskalenko (2017), Moskalenko and McCauley (2009) and McCauley and Moskalenko (2011) for which there is already strong empirical backing (Gøtzsche‐Astrup, 2018). The TPM was largely developed to be consistent with psychological models that deal with attitudinal and behavioral outcomes as they relate to the same object or action. As noted above, when seeking to examine the attitudinal antecedents of behavior, it is important that the attitude being examined achieves a high level of specificity with respect to the associated behavior (Fishbein and Ajzen, 1975). As such, the TPM's radical opinions period, or cognitive radicalization pyramid, is constructed in reference to the outcomes of the “radical actions” pyramid.

Starting with the radical behaviors pyramid, activism and radicalism are differentiated from each other primarily in that the former generally consists of legal, nonviolent ideologically motivated behaviors, and the latter generally consists of illegal and violent ideologically motivated behaviors. With respect to the latter, however, these behaviors are essentially still subterroristic in nature, with terrorism being the targeted use of ideologically motivated lethal violence (McCauley & Moskalenko, 2017).

Behaviors are in theory much easier to categorize than attitudes, however, which are more hypothetical constructs. Following Ajzen's (1988, p. 4) definition of an attitude as being “a disposition to respond favorably or unfavorably to an object, person, institution, or event,” and like criminal attitudes towards violence (Fincham, Cui, Braithwaite, & Pasley, 2008; Nunes, Hermann, Maimone, & Woods, 2015), radical attitudes should be assessed by a high level of specificity with respect to an object (e.g., terrorism), person (e.g., Osama bin Laden), institution (e.g., Al‐Qaeda), or event (e.g., 9/11) Borum, 2015; Schmid, 2017). Indeed, even before the development of the TPM, scholars were already assessing cognitive radicalization from responses to questions assessing support, acceptance, or justification of these items. And whilst imperfect, these are still the best measures we have for cognitive radicalization (Schmid, 2017). Radical attitudes can, therefore, be assessed by examining support, justification, or acceptance of radical behaviors in a general sense, or with respect to others engaging in such behaviors.

This distinguishes the attitudinal outcome from the intentional outcome, which the TPM calls “personal moral obligation,” in which an individual feels, or expresses their feelings that they should engage in radical behaviors. Along the spectrum of intentions, and with respect to radicalization specifically, there is a close relationship between desire, readiness, willingness, and behavioral intentions (Brynielsson et al., 2013). Indeed, based on the TPM framework, Moskalenko and McCauley (2009) created the Activism‐Radicalism‐Intentions‐Scale (ARIS), an instrument used to assess intentions toward engagement in activist or radical actions and behaviors. This tool has been used in a number of studies to assess radical intentions and is growing in popularity. However, given the sensitivity of asking study participants about their intentions toward illegal behaviors, some researchers have adapted the ARIS to assess attitudes. For example, instead of asking respondents if they would engage in a specific radical behavior, they ask them to what degree they support, or agree with someone else who engages in that behavior (e.g., Ellis et al., 2016).

**Figure 1:**
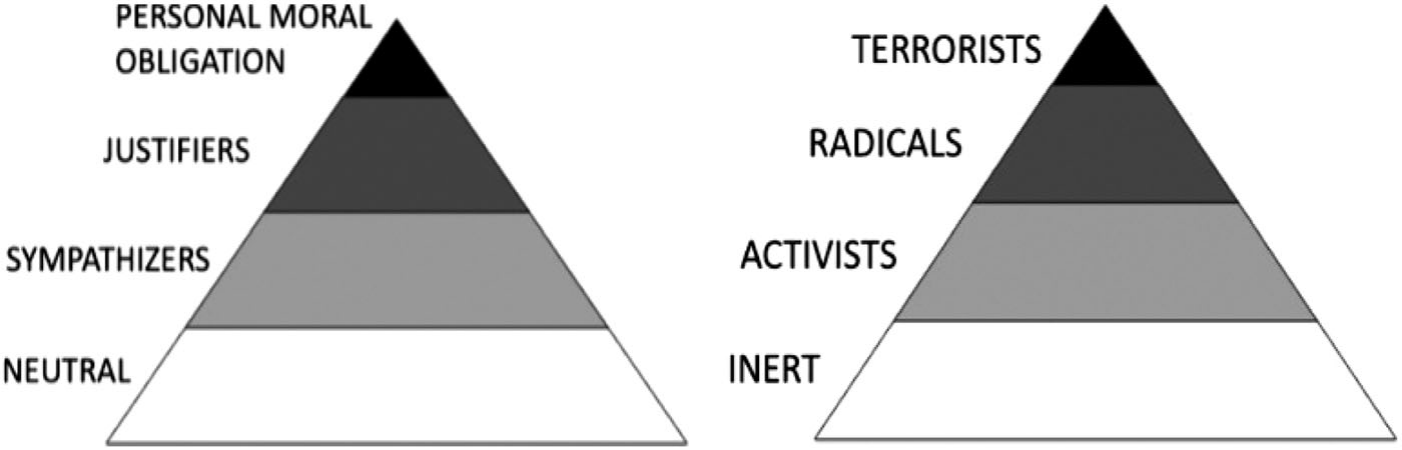
McCauley and Moskalenko's (2017) two‐pyramid model

**Figure 2:**
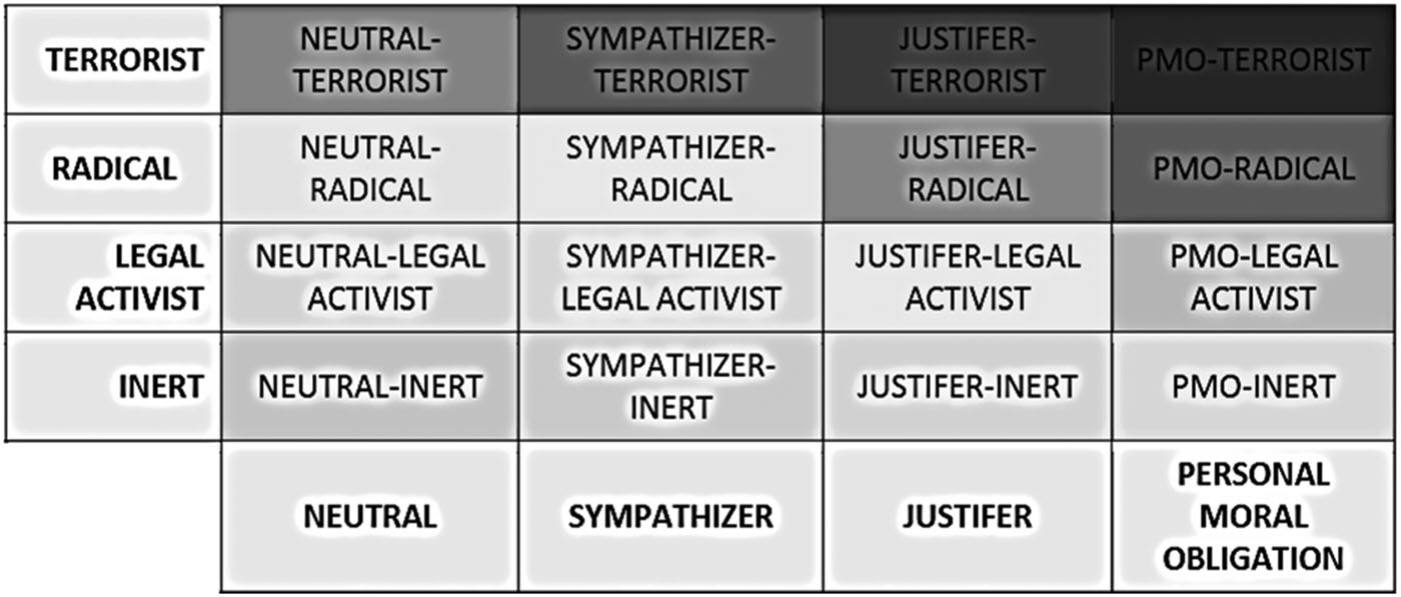
Possible beliefs‐actions combinations (Orlina & Desjardins, 2012, p. 16)

Unlike other frameworks, the TPM is nondirectional and does not specify any particular direction to the move from and between its different levels nor does it require passing through one level to progress to the next. The TPM thereby provides for the possibility that terrorists need not be exceptionally radicalized, and pathways that lead to nonviolent outcomes, accepting that most people who hold radical views will never turn to violence (Borum, 2011a, 2011b). According to the model, every individual "radical" exists at some level on both pyramids simultaneously. The narrowing shape of the pyramid at each subsequent level indicates that a smaller population displays the outcome. For example, while there may be 5% or more of a given sample that are “justifiers” of terrorism, a much smaller percentage are likely to believe they have a personal moral obligation to carry it out (Leuprecht, Hataley, Moskalenko, & McCauley, 2010). Even among those who hold that they have a personal moral obligation, or express intentions or a willingness to engage in violent radical behaviors, the majority will remain forever inert. However, whilst there are exceptions, it can be assumed that the majority of terrorists did at some point prior to their actions, hold that they had such a personal moral obligation. Nevertheless, these represent the smallest proportion of the population of radicals. (Figures 1 and 2).

There are many advantages to a typological and outcome‐based framework such as the TPM as part of a systematic review and meta‐analysis. First, it provides the type of definitional clarity and consistency that is needed for operationalization, whilst simultaneously reflecting the approaches taken by organizations such as the EU and the general logic underpinning counter‐radicalization initiatives more generally. For this reason, the TPM, and pyramid models in general, have also become popular in counter‐radicalization and counter‐terrorism interventions research (Orlina & Desjardins, 2012). Second, it sets clear categorization standards that exclude distantly related beliefs that may provide important comparisons but detract from the object of interest.

The TPM does not, however, provide a set of mechanisms or explanation as to why some may move from opinions to actions, or attitudes to behaviors, and also why some who may feel a personal moral obligation will remain forever inert. But the researchers who developed the TPM have offered some possible explanations, in the form of different risk and protective factors. For example, personal or group grievances, or thrill‐seeking may be risk factors for the move from beliefs to actions, and parental bonds may inhibit it (Leuprecht et al., 2010; McCauley & Moskalenko, 2008; Moskalenko & McCauley, 2011). Indeed, the entire relationship between attitudes, intentions, and behaviors as it pertains to criminal and criminal‐analogous outcomes, is mediated by risk and protective factors (Tuck & Riley, 1986). That is, the presence or absence of risk and protective factors can explain why a small percentage of individuals with radical attitudes and/or intentions will eventually engage in radical behaviors, whilst most would not (Malthaner, 2017; Stern, 2016).

### 1.3 Overlaps between criminal and radical attitudes, intentions, and behaviors: a risk and protective factor framework

The risk‐protective factor framework is a probabilistic, rather than a deterministic one. That is, while risk factors can be found to predict a range of criminal and criminal‐analogous outcomes, such as gang involvement (Higginson et al., 2015; Hill, Howell, Hawkins, & Battin‐Pearson, 1999) or general violent offending (Herrenkohl et al., 2000), most individuals possessing even all the most important risk factors will never actually offend. Risk factors only increase the propensity to offending and do not predict offending as a given. However, they can help to identify and differentiate the types of individuals at greater risk (Kazdin, Kraemer, Kessler, Kupfer, & Offord, 1997; Murray & Thomson, 2010; Shader, 2001) and which risk factors and the degree of their presence differentiate between different types of offending outcomes (Esbensen, Peterson, Taylor, & Freng, 2009; Horgan, Shortland, Abbasciano, & Walsh, 2016). Similarly, protective factors can cancel out or override the effects of risk factors (Farrington, Ttofi, & Piquero, 2016). As such, it is the likelihood that an individual will hold on to certain criminal attitudes, or engage in criminal behaviors, that will be increased or decreased by the cumulative and interactive weight of risk factors over protective factors, or vice versa ( Folk et al., 2018; Lösel & Farrington, 2012).

In criminology, it has long been found that criminal attitudes and criminal intentions are among the best predictors of criminal behaviors (Gendreau, Little, & Goggin, 1996; Simourd, Hoge, Andrews, & Leschied, 1994). However, not all measures of criminal attitudes are equal. For example, Fincham et al. (2008) found that a revised version of an interpersonal violence attitudes scale had greater predictive value for actual interpersonal violence behaviors than a more general version. Nunes et al. (2015) found that support for the use of violence is a distinct type of criminal attitude and that it is a better predictor of violent behavioral outcomes than general criminal attitudes. Traditional criminogenic factors such as deviant peers (e.g., Megens & Weerman, 2010), low self‐control (Walters, 2016) are known moderators of the attitude‐behavior consistency for an ordinary crime. There is evidence that similar risk factors may mediate the relationship between radical attitudes and behaviors (Bélanger, Caouette, Sharvit, & Dugas, 2014; Kruglanski et al., 2018). Given these similarities, and a growing body of evidence pointing to overlaps between terrorism and crime, terrorists and criminals (e.g., Clarke & Newman, 2006; LaFree & Dugan, 2004; Mullins, 2009), and the risk and protective factors for criminality and radicalization (e.g., LaFree, Jensen, James, & Safer‐Lichtenstein, 2018; Lösel, King, Bender, & Jugl, 2018), it makes sense to approach the study of risk and protective factors in a similar fashion (Borum, 2015).

### 1.4 A systematic review of putative risk and protective factors: taking a “field‐wide approach”

As described above, risk and protective factors are mediators of the attitude‐intention‐behavior continuity. However, they also directly affect attitudinal, intentional, and behavioral outcomes in and of themselves. It is a research imperative to “tease out the often subtle and complex interactions between attitudes and non‐belief‐related factors” (P. R. Neumann, 2013, p. 880). This is only possible by organizing and comparing the magnitude of the effects between belief and non‐belief‐related factors, including across‐related outcomes.

This systematic review follows an epidemiological approach and will attempt to present a “field‐wide meta‐analysis of observational associations” regarding putative risk and protective factors for the different outcomes of radicalization. Unlike traditional reviews that focus on a specific factor, type of factor, or set of factors (e.g., education, mental health), the field‐wide approach seeks to collect and organize the evidence for an unspecified array of factors, allowing the literature to determine what will be included, rather than the researchers. Many reviews from criminology and psychology take such an approach, even if they do not explicitly refer to it as such. This approach seeks to "consider all data on all assessed risk factors for the outcome of interest and to compare the relative availability of data for each of the putative risk factors across the respective studies" (Serghiou, Patel, Tan, Koay, & Ioannidis, 2016, p. 59).

The field‐wide approach used by this review offers a number of potential benefits. First, it enables the identification of factors that are not as frequently considered as others. Second, it enables the assessment of the relative magnitude of factors. Third, it enables a comparison of the different factors and their magnitudes across a range of outcomes for the phenomenon under inquiry. Through the use of meta‐analytic techniques, this approach can also identify differential effects for factors in different contexts (e.g., US and the EU), as well as between different radicalizing doctrines (e.g., right‐wing, left‐wing, religious, etc) (Jensen, Atwell Seate, & James, 2018; Monahan, 2012, 2017). Given that there is great heterogeneity among violent offenders in general, and “one‐size‐fits‐all” solutions are inappropriate for this subfield of criminology as well (Widom, 2014), these benefits will enable the production of the type of evidence needed to assist in avoiding such pitfalls.

### 1.5 Why it is Important to do the review

As noted above, despite the growth in terrorism research, it continues to be lamented that empirical study accounts for only a small percentage of terrorism research, especially relating to risk factors (Christmann, 2012; Lum et al., 2006; Silke, 2008). This gap in the research has left it to policy makers to develop policies and strategies that are not grounded in evidence and which are based on untested theoretical assumptions and political considerations (Neumann & Kleinmann, 2013; Sageman, 2014; Victoroff, 2005). It seems that we do not actually know much about what risk factors should be being examined to assess the risk of future radical behaviors, or which should be targeted in order to reduce the risk of future radical behaviors, namely because we do not know what the risk and protective factors are (Scarcella, Page, & Furtado, 2016). Among the evidence that does exist are mixed and often contradictory findings (Allan, Glazzard, Jesperson, Reddy‐Tumu, & Winterbotham, 2015; Bondokji, Wilkinson, & Aghabi, 2017; Victoroff, 2005). We also know very little about the relative magnitude and clustering of risk and protective factors. That is, we do not know whether, for example, unemployment is more or less important than education (Crenshaw, 2007; Gill, 2016; Hafez & Mullins, 2015; Haggerty & Bucerius, 2018; Staring, 2014).

Recently Scarcella et al. (2016) conducted a systematic review on methodologies used to perform risk assessments of individual radicalization. They found that for many of the tools used, even those that apparently take a more evidence‐based approach (e.g., Extremism Risk Guidelines [ERG22+]), the risk factors they included were not evidence‐based. On account of this, and related to the issue of absent and mixed evidence, most tools reviewed used a nominal scaling method, in which the presence or absence of each factor is given an equal weighting (Klausen, Campion, Needle, Nguyen, & Libretti, 2016). There are two dangers inherent in risk assessments used to predict future radical behavior based on misidentified or misspecified factors. First is the failure to properly identify those most at risk, which could result in a terror attack. Second is the false identification of those who pose little or no risk, which could lead to impinging on the rights of innocents, which also entails wide‐reaching social implications (Scarcella et al., 2016). Third, when used in the context of targeting dynamic factors in order to reduce the risk of future radical behavior, as in the context of the Risk‐Needs‐Responsivity approach, a poor identification of specific needs is likely to lead to unsuccessful intervention outcomes (Dean, 2016; Mullins, 2010).

To date, the only Campbell Collaboration systematic review that has been identified as being somewhat related to the current topic is Lum et al.'s (2006) review on counter‐terrorism policies. There have however been a few non‐Campbell reviews conducted on radicalization more generally; however, they either only refer to risk and protective factors in passing, or have otherwise been unable to provide any sort of quantitative synthesis of the evidence and (e.g., Christmann, 2012; Madriaza & Ponsot, 2015; McGilloway, Ghosh, & Bhui, 2015; Munton et al., 2011; Vergani, Iqbal, Ilbahar, & Barton, 2018). Some recent reviews have focused on specific factors, such as mental health (Misiak et al., 2019), and the Internet (Hassan et al., 2018); however, they too have been unable to provide any quantitative synthesis. To date, no review or meta‐analysis specifically on the topic of risk factors in radicalization and recruitment to terrorism.

The current review represents an important first step toward developing knowledge and understanding concerning what the putative risk and protective factors are, what their relative magnitude is. This will, of course, represent only a starting point in enabling the development of more scientifically based and effective interventions (Blum & Ireland, 2004; Borum, 2015; Piquero, Farrington, Welsh, Tremblay, & Jennings, 2009).

Beyond the direct contributions to the literature that this review will ultimately provide, the results of this review will also be utilized in the development of inputs for agent‐based modeling simulations that are being conducted by the reviewers as part of project PROTON, a Horizon 2020 funded project on the processes of radicalization and recruitment to terrorism. This project includes direct involvement and cooperation of leading policy makers from the European Union. Therefore, the results of this systematic review will play an important role in informing policy makers in the context of the development of evidence‐based policies.

## 2 OBJECTIVES

The primary objectives of this systematic review are to provide information that can help in answering important questions regarding the risk and protective factors associated with radicalization outcomes. As a field‐wide review, the first objective is to identify what the different individual‐level, putative risk, and protective factors are for which empirical evidence exists. The second objective is to categorize, organize, and arrange the factors in a series of rank orders according to their identified effect sizes in order to assess the relative importance of the different factors. The third objective is to identify areas of overlap and divergence in the effect sizes for the different factors across outcomes, and also between variables such as region (e.g., US and the EU) and ideological association (e.g., right‐wing, left‐wing, religious, etc). In summary, through the use of meta‐analytic techniques, this review seeks to address the following questions.

1. What are the risk and protective factors associated with cognitive radicalization?
2. What are the risk and protective factors associated with behavioral radicalization?
3. What are the shared and differentiating risk factors for the different outcomes of radicalization?
4. What are the relative magnitudes of the effect sizes for the different factors across the different outcomes?
5. What are the sources of heterogeneity between factors as they pertain to different ideologies and radical doctrines (e.g., right‐wing and Islamist inspired), and to different regions (e.g., EU and the US)?
  In addition, the review's secondary objectives are as follows.
6. To provide guidance for a secondary systematic review that will focus on antiradicalization interventions and how risk factors can be mitigated.
7. To provide data and inputs in the form of effect sizes to be used in the development of agent‐based modeling.

## 3 METHODOLOGY

### 3.1 Criteria for including and excluding studies

#### 3.1.1 Types of studies

The literature is heavily characterized by studies representing theoretical discussions or literature reviews, with only a small percentage of studies being empirically based (Christmann, 2012; King & Taylor, 2011; Sageman, 2011; Silke, 2008), and an even smaller percent being quantitative (Neumann & Kleinmann, 2013; Schuurmann, 2018). This review seeks to extract only quantitative studies and excludes qualitative studies, including studies that are purely theoretical, provide theoretical models, literature reviews, opinion pieces, and those studies based on basic descriptive statistics. Given the nature of the review, we will collect data from studies employing case‐control, single‐sample longitudinal, and single‐sample cross‐sectional designs (see eligibility criteria below).

As Jolliffe, Murray, Farrington and Vannick (2012) explain, when conducting systematic reviews on risk factors—as opposed to interventions—there is a need, or at least a justification, for lowering the number of variables relating to the inclusion quality threshold. As such, with regards to these observational studies, the review will only include those studies which have an *N* of >50. With regard to the sampling methodology, a wide range of methods will be considered acceptable as long as they provide a reasonable basis for making inferences to the intended population of the review.

#### 3.1.2 Dependent variables

In order for a study to be included in the review, the dependent variable(s) must be in line with at least one of the top two tiers of each of the pyramids in the TPM. For cognitive radicalization, this includes studies whose dependent variable(s) assess support for, justification of, or a willingness or intention toward the commission of radical violence or terrorism. The literature includes the use of some validated instruments for assessing these outcomes. Some examples of the most well‐known tools are listed and described in Table 1. Studies utilizing any of these or similar instruments, or adapted versions of these or similar instruments, will be eligible for inclusion in the review.

**Table 1:** Examples of instruments used to measure cognitive radicalization

Instrument name	Description	Example of usage
Sympathy for violent radicalization and terrorism (K. Bhui et al., 2014)	A 16‐item tool measuring sympathies and condemnations of radical behaviors from violent protest to suicide bombings	Rousseau et al. (2019)
Activism‐Radicalism‐Intentions Scales (Moskalenko & McCauley, 2009)	10‐item tool assessing intentions to engage in legal and illegal forms of activism	Decker and Pyrooz (2019)
Pro‐Violence and Illegal Acts in Relation to Extremism Scale (Ozer & Bertelsen, 2018)	A six‐item tool using a seven‐point Likert scale	Ozer (2020)
Militant‐Extremist Mindset Pro‐Violence Subscale (Stankov, Higgins, Saucier, & Knežević, 2010)	A 10‐item subscale using a five‐point Likert scale	Vergani, O'Brien, Lentini, and Barton (2019)

In addition, studies using other single‐ and multiple‐item measures will be eligible for inclusion when they are found to be in line with the above‐noted outcomes of the TPM. These measures may measure sympathy, support, or justification of general forms of radical violence, or may refer to specific types of radical violence (e.g., suicide bombings), or specific events of radical violence (e.g., 9/11, 7/7 bombings). Some examples are listed in Table 2.

**Table 2:** Examples of nonvalidated measures of cognitive radicalization

Measure	Assessment	Usage
Some people think that suicide bombing and other forms of violence against civilian targets are justified in order to defend Islam from its enemies. Other people believe that, no matter what the reason, this kind of violence is never justified. Do you personally feel that this kind of violence is often justified to defend Islam, sometimes justified, rarely justified, or never justified?	Four‐point scale: 1 = often, 4 = never	PEW report (see McCauley, 2012; Victoroff, Adelman, & Matthews, 2012)
What is your attitude towards the murder of Theo van Gogh, in November 2004?	Five‐point scale: 1 = very negative, 5 = very positive	Doosje, Loseman, and Van Den Bos (2013)
Some people said that the July (7/7) bombings were justified because of British support for the US war on terror. Do you agree?	Four‐point scale: 1 = strongly disagree, 4 = strongly agree	Tausch, Spears, and Christ (2009)

For behavioral radicalization, this includes studies whose dependent variable(s) assess involvement in or commission of radical violence or terrorism offenses. For the most part, we expect that studies will measure having engaged in radical violence or terrorism offenses as a dichotomous variable (e.g., LaFree et al., 2018). However, when studies assess self‐reported involvement in radical violence they may employ an ordinal or interval measure that combines a variety of self‐reported behaviors (e.g., Pauwels & Schils, 2016).

#### 3.1.3 Controls/sample

In order for a study to be included in the review, the sample must include a control or comparison group that does not display the outcome of interest. That is, a study's sample must display variation on the dependent variable. For example, in a cross‐sectional or longitudinal study examining support for terrorism, the sample must include respondents or participants who do not support terrorism. Similarly, in a case‐control study containing a sample of terrorists, it must also include a sample of nonterrorists or nonviolent terrorists.

#### 3.1.4 Population

The review will include studies whose samples are made up of individuals. The review sets no limitations on the population of a sample based on the age, sex, race, or religion of the sample or those included in it. The review sets no limitations based on the ideological doctrine associated with the inquiry of the study being assessed for inclusion and will include studies that assess radicalization outcomes across the entire spectrum of guiding doctrines (e.g., religious, right‐wing, left‐wing, ethno‐nationalist and single issue, etc). As detailed ahead (see Section 3.1.7******), populations will only be limited by the geographic locations from which they are drawn.

The populations that will be included will, therefore, include terrorists, behavioral radicals, cognitive radicals, and individuals not displaying these outcomes who form part of the larger control or comparison portion of the sample.

#### 3.1.5 Types of risk and protective factors

According to Kraemer et al. (1997), for a factor to be classified as a risk factor, it must demonstrate a predictive quality and also be shown as having been present prior to the outcome (e.g., radicalization, recruitment, or terrorism). Theoretically, only single‐sample longitudinal studies can establish the temporal ordering of factors, namely that an independent predictor preceded the outcome temporally (Murray, Farrington, & Eisner, 2009). This means that even for case‐control or cross‐sectional studies, even when employing regression techniques that may produce results showing a predictive quality of a given factor, temporal ordering is still absent. Nevertheless, when a factor is found to correlate with the outcome of interest in the theorized direction, we can classify it as a “putative factor” (Kraemer et al., 1997, Kraemer, Stice, Kazdin, Offord, & Kupfer, 2001). Such a classification is common in psychology (e.g., May & Klonsky 2016), criminology (Assink et al., 2019), and radicalization research (K. S. Bhui, Hicks, Lashley, & Jones, 2012; Lloyd & Dean, 2015; Monahan, 2012).

For the purposes of the current study, we refer to all factors that display a positive or negative correlation with one of the included outcomes of interest as being a “putative risk/protective factor,” respectively, even though some factors could be characterized as risk factors (Jacobi, Hayward, de Zwaan, Kraemer, & Agras, 2004). As such, we derive effect sizes for such factors, as described below (see Section 3.8****), from case‐control, longitudinal, and cross‐sectional studies using a combination of the correlation matrices, descriptive factors, and/or partial effect sizes/coefficients from regression models.

As a field‐wide review, no specific factors are specifically sought and no predeterminations will be made as to which factors will be identified, and as such does not include any outcome labels in its search terms (Murray et al., 2009; Serghiou et al., 2016). We will include all individual‐level factors, including both static and dynamic factors, that can be said to fall under domains such as, but not limited to: socio‐demographic, experiential and background, social, psychological, economic, socio‐economic, socio‐psychological, environmental, or push, pull, and personal factors. Based on our initial examination of the literature, the below list provides some of the most commonly discussed and studied factors, and those that are likely to be included the review (this list is not exhaustive nor should it be taken to be representative of what will feature in the final review).

##### Individual and experiential characteristics

- Age, gender, education, immigrant status, marital status, criminal history, drug and alcohol use, victimization, encounters with police and procedural justice, trauma, and so forth.

##### Social factors

- Discrimination, marginalization, ostracism/isolation, societal connectedness, previous criminal history, poor employment history, family issues, immigrant status, Religion, community, discrimination, political grievances, policing and counter‐terrorism, access to social services such as education and healthcare, education level, media influence, peer groups, Collective efficacy, legitimacy, and so forth.

##### Economic factors

- Unemployment, real poverty, perceived poverty, economic disparity, real and relative deprivation, wage gap, income level, individual socio‐economic class, meso‐level (community level) economic situation, property ownership/housing status, welfare situation, and so forth.

##### Psychological factors

- Mental illness, depression, anxiety disorder, narcissism, neurological problems, slow motor development, lack of empathy, ideological mentality, utopian/messianic worldview, dualistic mentality, personal grievance, personality traits (e.g., low self‐control, anger, hate, authoritarianism/fundamentalism), and so forth.

#### 3.1.6 Measurement of outcomes and risk/protective factors

As described above, the review will include studies whose measurement of both outcomes and factors are dichotomous, ordinal, discrete, or continuous, and which will be based off of a range of instruments and forms of coding. Measures of outcomes and factors will be derived from self‐reported, family reported, government reported, law‐enforcement reported, practitioner reported, and open‐source database‐generated data.

The review will employ the appropriate statistical conversions in order to enable the grouping of effect sizes based on these different measures into single analyses.

#### 3.1.7 Types of settings

Systematic reviews on risk factors for behavioral outcomes, such as violence, delinquency, and gang involvement, are often examined separately between high‐income and low‐middle income countries. The rationale is that the macrolevel settings and contexts are too dissimilar and as such, issues of heterogeneity would mask the true effects. As such, there is great methodological value to narrow risk factor systematic reviews to specific types of contexts (Higginson et al., 2015; Murray et al., 2018). In addition, there may be added methodological value in the separating of searches based on country and country type, including with regard to the databases that should be searched and also with respect to the synthesis of the data identified (Shenderovich et al., 2016).

It is often pointed out in the literature that the “driving factors” of terrorism are quite different between democratic and nondemocratic countries. In addition, trends in terrorism, as well as radicalization processes appear to be quite different between these different regime types (GTI, 2016). Taking such issues into consideration, some researchers have recognized the importance of examining democracies and nondemocracies separately with regard to terrorism‐related studies, since aggregated analyses often produce confounding results. Similarly, some researchers examine terrorism and violence‐related issues separately between high‐income and low‐income countries (e.g., Enders & Hoover, 2012). While both these approaches have methodological value and a similar theoretical underpinning, this review has chosen to the former approach, since democratic countries represent open societies, coupled with economic prosperity, and therefore provide similar societal (macro) and community level (meso) contexts. In addition, terrorism in democratic countries appears to be more prevalent and more stable. Furthermore, the recent rise in the threat of terrorism in such countries demands further development of our understanding risk factors leading to radicalization that are specific to such places.

Since there is no established preference as to whether reviews should separate by income or regime type, in this review, we seek to bridge these approaches by limiting our inclusion to democratic countries that are also Organization for Economic Co‐Operation and Development (OECD) countries. As such, the Democracy Index was cross‐matched with the OECD countries, resulting in the inclusion of all 37 OECD countries with the only exclusion being Turkey. In order for a study to be included in the review, the majority of its sample must come from at least one of the included countries, and the country must have met the criteria for inclusion in this list at the time at which the study was carried out, or at the time period in which the sample was collected from (Table 3).

**Table 3:** List of included countries

Australia	Czech republic	Greece	Japan	Netherlands	Slovenia
Austria	Denmark	Hungary	Korea	New Zealand	Spain
Belgium	Estonia	Iceland	Latvia	Norway	Sweden
Canada	Finland	Ireland	Lithuania	Poland	Switzerland
Chile	France	Israel	Luxemburg	Portugal	UK
Columbia	Germany	Italy	Mexico	Slovakia	USA

#### 3.1.8 Languages

Primary searches will be conducted in English as the overwhelming majority of relevant studies will at least be indexed in English and/or have abstracts in English. Supplementary searches will also be conducted in Dutch and German as relevant research that may not have been translated is known to exist. Relevant studies that are found to be published in any other language will be sent for translation.

#### 3.1.9 Exclusion criteria

The following types of studies will be excluded.

1. Studies examining radicalization and recruitment in nondemocratic countries.
2. Studies examining general political radicalism/political extremism as generally assessed by; support for specific types or sets of legitimate policies, such as those pertaining to immigration, or crime and punishment, or legal political parties running in elections (including using voting for legal parties as a proxy).
3. Studies examining radicalization by way of analyzing antidemocratic attitudes, anti‐western attitudes, or racist, xenophobic, or anti‐semitic attitudes as a proxy for radicalism.
4. Studies examining general violence and racially motivated violence.
5. Studies examining predictors of the occurrence of terrorism events.
6. Studies examining differences between activities or events between groups or countries.

#### 3.1.10 Relevance decisions

The first stage of the review process will consist of two trained reviewers conducting searches and making initial judgments regarding study suitability and inclusion based on title and abstract reviews. In addition, the following will be considered.

1. An extraction sheet (excel format) will be used and completed for a study that passes the first threshold based on title and abstract. During data extraction, a quality/inclusion checklist will be used (see Supplorting information materials).
2. An assessment tool to check for reviewer consensus will be used. Where a study has been selected by only one of the reviewers, it will subsequently be checked by a senior reviewer where the full text of the article will be reviewed and the senior reviewer will then present their decision to the two reviewers.
3. In order to minimize reviewer inclusion bias, senior reviewers will analyze a sample of 5% of included studies and check for rater reliability.

### 3.2 Search strategy

The review will search for both published and unpublished literature based on the above inclusion criteria. The search terms have been developed in order to increase the likelihood that the most relevant studies will not be missed and so that the literature on risk factors will be broadly covered. Specifically, there is a delicate balance between sensitivity and specificity, and this varies greatly between different databases. It was decided that none of the outcomes would be part of our search terms in order to avoid limiting the number of relevant studies that may come from other fields, such as psychology. Our familiarity with the current body of literature has also been the basis for the decision to utilize the range of different terms that are used to describe radicalization and recruitment to terrorism and which can often be found in the different literatures that study these issues. As has been noted in the literature on systematic reviews, reviews dealing with risk factors may have limited search tiers due to the need to balance sensitivity and search precision (Hammerstrøm, Wade, Hanz, & Jørgensen, 2009; Shenderovich et al., 2016). Following is a list of our four search tiers.

1. Radical* OR recruit* OR extrem* OR violent extrem* OR violent radical* OR foreign fighter* OR Terror* OR Lone wol* OR lone‐wol* OR homegrown OR home‐grown OR sympath* OR support OR OR Justif* OR facilitate OR engage* OR activis*
2. Jihad* OR Islam* OR Salaf* OR right‐wing OR neo‐Nazi OR far‐right OR nationalist OR white‐supremacist OR left wing OR extreme left OR anarchist OR single‐issue
3. *Risk* OR *factor* OR predict* OR propensity OR likelihood OR predispose* OR predisposition OR vulnerab* OR causal OR putative OR determinant OR Root OR correlate*
4. *Democra* OR West* OR Europ* OR "country name"

Search strings will be applied on the databases mentioned in Table 4.

**Table 4:** Databases in which searches will be performed

Campbell library Cochrane Library DARE ERIC ISI Science/Social Science Citation Index PsycINFO PubMed Medline Journal of Deradicalization	Google Scholar Social Science Research Network e‐library (SSRN) Social Care Online Sociological abstracts BRÅ NCJRS (National Criminal Justice Reference Center) Bibsys START Perspective on Terrorism

In addition, as this review is being carried out as part of a larger research project, we will include relevant studies being conducted by members of the research consortium. We expect that at least 1–2 of these studies will fit our inclusion criteria. In addition, whilst searches are ongoing, leading researchers and research institutions will be contacted in an effort to try and identify unpublished research and gray literature. We will also share the protocol and the list of included studies at the time contact is made with these researchers to better assist them in assessing whether they are aware of any missing studies that would meet the inclusion criteria.

### 3.3 Pilot search

Based on the above‐mentioned inclusion and exclusion criteria, pilot searches were conducted on PsychInfo (Ovid). This search engine was chosen since it is known to contain essential studies pertaining to radicalization. We carried out a number of pilot searches to test the balance of sensitivity and specificity of our search strings. To ensure that our search strategy was capturing the studies of relevance, we carried out multiple search combinations. One example pilot search utilized a two tier search strings “((radicalization or radicalization or radical or terrorism) and (risk factor or risk or vulnerability or association or associated)).ti. and (radicalization or radicalization or radical or terrorism).ab. and (risk factor or risk or vulnerability or association or associated).ab,” where only titles and abstracts were searched using the search filters for titles and abstracts. An initial 82 results were returned from this search. Based on a skimming review of the citations and abstracts, the four studies passed to the extraction phase. Subsequently, two of these studies were excluded based on criteria pertaining to their settings (Table 5).

**Table 5:** Pilot search excerpt

Reference	Study design	Incl/Excl
Elbakidze, L. & Jin, Y. H., Are economic development and education improvement associated with participation in transnational terrorism? *Risk Analysis. Vol. 35(8), 2015, pp. 1520‐1535*	*Cross‐sectional*	*Excluded based on dependent variable*
Najeeb Shafiq, M., & Sinno, A. H. (2010). Education, income, and support for suicide bombings: Evidence from six Muslim countries. *Journal of Conflict Resolution*, *54*(1), 146‐178	*Cross‐sectional*	*Excluded based on country*
Bhui K, Warfa N, Jones E., Is violent radicalisation associated with poverty, migration, poor self‐reported health and common mental disorders? *PLoS ONE 9(3), 2014*	*Cross‐sectional*	*Included*
Krueger, A. B. (2008). What makes a homegrown terrorist? Human capital and participation in domestic Islamic terrorist groups in the USA. *Economics Letters*, *101*(3), 293‐296	*Case‐control*	*Included*

### 3.4 Description of methods used in primary research

Based on the reviewers' familiarity with the current body of literature, including from a wide range of disciplines, we expect that the majority of studies will be single‐sample cross‐sectional studies examining different outcomes associated with cognitive radicalization. These studies, together with the odd longitudinal study, generally provide descriptive statistics, bivariate correlations, as well as multivariate models employing different regression techniques and path analyses. The majority of these studies are expected to be based off of self‐reported measures.

Case‐control studies, which may compare cognitive or behavioral radicals (e.g., terrorists) with a sample of those not displaying the outcome, may provide descriptive statistics, bivariate correlations, null‐hypothesis tests such as *t* tests, χ^2^ tests, *F* tests, as well as multivariate models employing different regression techniques. Most of these studies are expected to be based off of open‐source based databases which have been constructed from a variety of sources. While control group samples may also be derived from similar sources, they may also be based off of self‐reported measures or clinician‐reported measures.

### 3.5 Criteria for determination of independent findings

Our pilot searches have revealed that some studies base their analyses on the same secondary datasets (e.g., Pew Report, 2007). Other studies may report multiple statistical models. As such, in order to maintain independence of findings, a single report will only be allowed to provide one effect size for each risk factor. Where multiple effect sizes are reported for the same risk factor, an internal meta‐analysis will be conducted in order to obtain a single, pooled effect size (Higginson et al., 2014). A similar approach will be followed for studies based on the same original datasets published by the same authors since sometimes the same set of authors will conduct different studies based on the same datasets. As such, the reviewers will attempt to identify any overlaps of this nature and where possible will conduct an internal meta‐analysis and bias assessment. This assessment will be based on the completeness of the data and the risk of bias assessment of the studies, and all decisions will be reported in the final review.

### 3.6 Details of study coding categories

The data extraction and coding will be based on a code book that is made up of 70 items and which includes categories relating to publication information, methodology, study‐level characteristics, risk of study bias, the outcomes (risk and protective factors) included in the study, and the authors' conclusions (see Supporting Information Material). Following each set of 200 decisions, a random sample of 10 decisions (or 5%) will be chosen and intercoder reliability will be checked using *κ* statistical testing.

### 3.7 Assessment of methodological quality and risk of bias

We will use a risk of bias tool and apply it through a set of questions in the coding fields of the extraction tool shown in the Supporting Information Material (Higginson, Mazerolle, Ham Benier, & Bedford, 2014). Study quality assessment will be carried out by two trained reviewers. If there is disagreement, it will be mediated by a third member of the team, who will not be blind to the original quality assessment and who will make any final decisions. In the event that discrepancies arise in the coding stage, a senior reviewer will perform a review and make the final decisions.

### 3.8 Statistical procedures and conventions

This review will carry out a series of meta‐analyses, with a separate analysis being conducted for each factor for which two or more inputs, derived from two or more unique samples are identified. All effect sizes will be calculated as *r* and subsequently transformed to Fisher's *Z* in order to approximate a normal sampling distribution and achieve a more stable variance across different values (Borenstein, Hedges, Higgins, & Rothstein, 2009; Rosenthal, 1984). Fisher's *Z* will thus be used as the input for the meta‐analyses, as well as the statistic used for reporting of the outputs.

Studies such as the current review, which examine multiple factors and outcomes, generally use bivariate correlations for imputing effect size. Compared to effect sizes derived from multivariate models, bivariate effect sizes are more consistent and uncontaminated between studies (Hanushek & Jackson, 1977; Hunter & Schmidt, 2004; Pratt, Turanovic, Fox, & Wright, 2014). However, some studies may not provide correlation matrices, or sufficient descriptive data to enable the calculation of bivariate correlations. Instead, they may only report the results from a range of different types of multivariate regression models. In such cases, we will first contact the authors of the publication to attempt to acquire data needed to calculate bivariate effect sizes (Aloe & Thompson, 2013). Where such information is not forthcoming, we will standardize the partial effect sizes to be included as supplementary effect sizes (e.g., Higginson et al., 2014; Najaka, Gottfredson, & Wilson, 2001; Wong, Slotboom, & Bijleveld, 2010). While not ideal, this approach is preferable to conducting multiple separate meta‐analyses for each risk factor split by effect size measurement type, which would entail losing important data (Borenstein et al., 2009). In such instances, where there are more than two effect sizes from each category of derivation, metaregression will be used in order to assess whether the source of effect size derivation has an impact on the pooled estimate and the results will be reported (Aloe, Tanner‐Smith, Becker, & Wilson, 2016).

With regard to the calculation of effect sizes when no correlation matrix is available but the study includes adequate descriptive statistics, we will utilize the available information to calculate effect sizes using the appropriate formulas for the type of data present in accordance with Lipsey and Wilson's (2001) conventions by way of the "Practical Meta‐Analysis Effect Size Calculator" available through the Campbell Collaboration website.

With regard to the standardization of partial effects sizes, there are no standard conventions (Aloe & Thompson, 2013). As such, we adopt a number of widely accepted methods for each of the different types of measures that we anticipate will be encountered.

1. For linear regression models where the independent variable (IV) and dependent variable (DV) are both continuous, we calculate *r* as
  $$r=\frac{SD_{x}B}{SD_{y}}.$$
2. In situations in which standard deviations (SD) are not reported, or when the IV is dichotomous and the DV is continuous, or where the IV is ordinal or continuous and the DV is dichotomous, *r* will be calculated based on the *t ratio* (B/SE)
  $$r=t/\sqrt{t^{2}+}n-2.$$
3. In instances in which both IV and DV are dichotomous, and only *B* is reported, we will first calculate Cohen's *d* and then convert this to *r* as follows:

$$d=B\left(\frac{\sqrt{3}}{\pi }\right),$$

$$r=\frac{d}{\sqrt{4+d^{2}}},$$

where only the odds ratio (Exp. *B*) is reported, *r* will be calculated as

$$r=\frac{\text{OR}\frac{1}{2}-1}{\text{OR}\frac{1}{2}+1}.$$

Biostat's Comprehensive Meta‐Analysis (CMA) software (Borenstein et al., 2009) will be used for performing the meta‐analyses, including tests for publication bias, metaregressions, and moderator analyses. Random effects models will be used in order to deal with the expected homogeneity of the studies that will be included and which will also undoubtedly arise from the use of primarily correlational data from the types of observational studies that the review anticipates to include. In addition, risk factor effect sizes are often more heterogeneous than for interventions, in part because they are being examined among vastly different populations. For imputation, studies will be weighted based on their sample size. We will utilize the random effects estimator for τ^2^, the between‐study variance, that is preprogrammed in CMA V3, which is the Method of Moments approach of DerSimonian and Laird (1986).

### 3.9 Moderator analyses

The review will code a number of key study‐level characteristics: see Table 6.

**Table 6:** Study‐level characteristics

Variable	Description
Effect size derivation	Categorical: effect size derived from bivariate or partial effect size
Age	Continuous: mean age of the sample
Gender composition	Continuous: proportion of the sample that is male
Year	Continuous: last year of data collection
Ideology	Categorical: right‐wing, left‐wing, religious, and so forth
Region	Categorical: US, EU, other

Other possible study‐level characteristics for coding include: whether the outcome variable used a validated instrument, whether the risk/protective variable used a validated instrument, the measurement of the outcome or independent variable, whether a study was published or unpublished, and whether the study was in a language other than English.

A series of metaregression analyses will be carried out in order to assess the degree to which such factors may impact the results. Limitations for these analyses will be based on the number of studies included in each analysis, and whether, especially in the case of categorical variables, there are enough studies from each category in order to enable such analysis.

## CONFLICT OF INTERESTS

The authors declare that there are no conflict of interests.

## AUTHOR CONTRIBUTIONS

M. W. and B. H. contributed to the content. Y. L. and D. W. contributed to systematic review methods. M. W. and D. W. contributed to statistical analysis. M. W. and Y. L. contributed to information retrieval.

## Supporting information

Supporting information
